# Paperclip-Type Flexible Inductive Sensor Based on Liquid Metal Coils for Simple Fabrication and Multifunctional Applications

**DOI:** 10.3390/mi16080965

**Published:** 2025-08-21

**Authors:** Xun Sun, Kaixin Li, Zifeng Zhang, Linling Xiang, Yihao Zhou, Bin Sheng

**Affiliations:** 1 School of Optical Electrical and Computer Engineering, University of Shanghai for Science and Technology, Shanghai 200093, China; 2235052705@st.usst.edu.cn (X.S.); 233350670@st.usst.edu.cn (K.L.); 233350727@st.usst.edu.cn (Z.Z.); 233350619@st.usst.edu.cn (L.X.); 242200346@st.usst.edu.cn (Y.Z.); 2 Shanghai Key Laboratory of Modern Optical Systems, Engineering Research Center of Optical Instruments and Systems, Shanghai 200093, China

**Keywords:** paperclip-type helical coil, multifunctional inductive sensor, straightforward, simple, wearable devices

## Abstract

At present, high-resolution and reliable inductive sensors have increasingly emerged as a pivotal component in the advancement of flexible electronic devices. The integration of liquid metal with flexible substrates presents a promising approach for the fabrication of inductive sensors. This paper introduces a novel paperclip-type helical coil inductive sensor, characterized by advancements in both structural design and a simplified manufacturing process. The sensor comprises a fine silicone tube filled with liquid metal, encapsulated within polydimethylsiloxane (PDMS) glue. A significant innovation of this design is its complete elimination of the need for high-precision sacrificial metal molds. This approach bypasses complex processes such as precision mold machining, demolding, and post-mold residue cleaning, thereby significantly streamlining the production work-flow. We optimized the parameters of the paperclip-type helical coil, the aspect ratio, and the number of turns, achieving the maximum sensitivity under limited conditions. Experimental results demonstrate that this sensor is capable of tensile, pressure, and non-contact distance sensing. The linearity of the tensile sensing is exceptional ($R^{2}=0.999$), with consistent performance observed after 800 tensile cycles. The pressure sensing range extends from 0 to 230 kPa, and the non-contact distance sensing is effective within a range of 10 mm. Furthermore, the sensor exhibits strong performance in monitoring human physiological activities and metal distance detection, demonstrating significant application potential in flexible electronics and wearable devices.

## 1. Introduction

Flexible sensors are a great achievement in the field of modern sensing [1]. They have features such as flexibility, stretchability [2,3,4], wearability [5], and biocompatibility [6,7,8]. These sensors enable us to detect and acquire sensitive physiological signals without interfering with the body’s normal activities and are suitable for real-time monitoring of dynamic biological information. Flexible electronic devices are transforming various domains, including health monitoring [9,10,11,12], human–computer interaction [13,14,15], soft robotics [2,16,17], smart textiles [18,19], and electronic skin [20]. Currently, flexible electronic devices can be categorized based on their operational principles into resistive [14,21,22,23], capacitive [24,25], inductive [26,27], and optical path sensors [28], as well as sensors that monitor diverse quantities such as magnetic flux density [29]. Among these, resistive, capacitive, and inductive sensing technologies have garnered significant attention due to their compatibility with established circuit systems [30]. While resistive sensors offer high gauge factor (GF) values and robust anti-interference capabilities, their particle-based conductive networks suffer from contact instability, which diminishes repeatability [31,32]. Capacitive sensors, on the other hand, enhance repeatability by modulating signals through electrode geometric parameters, but they exhibit notable lag, limiting the precision of dynamic responses [33,34,35]. Hence, inductive sensors, characterized by their high resolution, accuracy, and reliability [36,37], are increasingly becoming central to the field of flexible sensors.

Inductive sensors facilitate high-precision measurement of physical quantities by leveraging the principle of electromagnetic induction. Typically, these sensors consist of coil structures composed of various conductive channels. According to pertinent literature, these coils can assume diverse configurations, including solenoid shapes [26,27,37], planar coil shapes [38,39,40], and 3D coils [41,42]. Xing et al. developed an inductive strain sensor by intricately winding non-ferromagnetic metal wires into a fiber coil structure analogous to a mechanical spring [37], enabling the identification of different object grasping actions through variations in inductance. Choi et al. developed a soft inductive coil spring strain sensor utilizing shape memory alloy coil springs [27], addressing the challenge of accurately measuring strain in spring beam actuators across a broad temperature range and in underwater environments. Traditional sensors composed of metal wires require insulating coatings to prevent short circuits; however, the uniformity and stability of these coatings can impact sensor performance consistency. Consequently, inductive sensors that integrate liquid metal with flexible substrates are increasingly becoming a focal point of research [43,44].

Among various conductive materials, liquid metals uniquely integrate the exceptional fluidity characteristic of liquids with the superior electrical conductivity inherent to metals [45,46], thereby rendering them increasingly significant in the domain of flexible devices. Recent studies have demonstrated innovative applications of liquid metals in this field. For instance, Zhou et al. utilized 3D co-printing technology to integrate liquid metal with a flexible substrate [47], creating a coaxial cable structure. This structure was subsequently configured into a spiral tube, enabling it to conform closely to cylindrical soft robots and facilitating high-precision measurement of tensile and bending deformations. Additionally, Wang et al. advanced the design of solenoids by fabricating silicone rubber microchannels via 3D-printed molds and injecting liquid metal [48], thereby enabling the production of strain-constant or ultra-sensitive coils. However, this inductive sensor manufacturing process, which depends on 3D printing molds for microchannel preparation, imposes stringent demands on printing accuracy and remains challenged by the high costs associated with high-precision instruments. Li et al. employed low-melting-point alloys as sacrificial materials to fabricate intricate channels within elastomers such as polydimethylsiloxane (PDMS) [41]. Following the injection of liquid metal and the subsequent removal of the sacrificial materials, they engineered three-dimensional curved high-density liquid metal coils. These coils were utilized in pressure sensing applications to propel biomimetic pufferfish robots and facilitate the movement of high-speed rotating robots. Despite circumventing the use of high-precision instruments to minimize costs, the process chain remains lengthy, and its stability is susceptible to disruption by residual materials. Recent literature on inductive sensors indicates that further exploration is required to develop a manufacturing process that is cost-effective, straightforward, and highly reliable.

This study presents the development of an innovative, multifunctional, flexible inductive sensor, characterized by a simple and straightforward production process. The sensor features a unique design, resembling a paperclip-type, thin-layer spiral structure. It is fabricated by encapsulating fine silicone rubber tubes filled with liquid metal using PDMS glue [49,50], capitalizing on the similar Young’s modulus of the silicone tubes and PDMS glue. Notably, this manufacturing approach circumvents the need for high-precision sacrificial metal molds. Instead, it leverages the high elasticity of silicone rubber tubes to serve as conductive channels, thereby simplifying the channel formation steps and reducing both the complexity and cost of the process. This study presents an innovative multi-functional flexible inductive sensor, distinguished by its highly simplified and direct manufacturing process. The sensor features a paperclip-shaped spiral coil design and is produced through a novel technique. Initially, a liquid metal-filled silicone tube is encapsulated, followed by encapsulation with PDMS adhesive. Notably, the silicone tube itself functions as a preformed self-insulating conductive channel, leveraging its intrinsic high elasticity. This approach effectively eliminates the necessity for complex and costly high-precision sacrificial metal molds or 3D-printed microfluidic templates commonly required in existing liquid metal channel formation technologies. Consequently, the channel formation steps are significantly simplified, leading to a substantial reduction in process complexity, material waste, and overall costs. The complementary Young’s modulus of the silicone tube and the surrounding PDMS matrix synergistically minimizes interfacial stress concentration during deformation. Simultaneously, the sensor exhibits tensile, pressure, and non-contact distance sensing capabilities. Experimental evaluations indicate that it maintains excellent linearity ($R^{2}=0.999$) at tensile strain and demonstrates significant consistency across 800 tensile cycles, thereby attesting to its reliability and durability. It responds to a broad pressure range from 0 to 230 kPa and performs effectively in non-contact distance sensing within a 10 mm range. Furthermore, the sensor’s practicality is evidenced by its ability to monitor human physiological activities, such as muscle contractions and elbow flexion, as well as its proficiency in distance sensing.

## 2. Materials and Methods

### 2.1. Materials

Polydimethylsiloxane (PDMS) and a silicone elastomer curing agent were purchased from DowCorning in Midland, MI, USA. A and B adhesives were mixed in a weight ratio of 10:1; the Young’s modulus was 1.5 MPa. The silicone rubber tubing employed in this study was sourced from Shanghai Xinrong Rubber and Plastic Technology Products Co., Ltd., in Shanghai, China, and its Young’s modulus is 2 MPa. The liquid metal Galinstan, composed of Ga:In:Sn in a ratio of 68.5:21.5:10 and with a melting point of 11 °C, was procured from Dongguan Dingguan Metal Technology Co., Ltd., in Dongguan, Guangdong, China. Additionally, the epoxy adhesive utilized was supplied by Dongguan Yihui Adhesive Co., Ltd., in Dongguan, Guangdong, China.

### 2.2. Preparation of the Sensor

**Figure 1:**
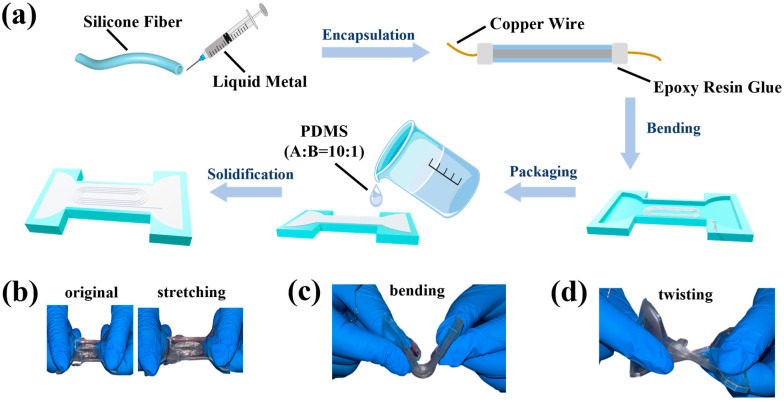
Preparation process and basic mechanical performance characterization of paperclip helical coil sensors. (**a**) Schematic of the fabrication process of the paperclip helical coil sensors. (**b**–**d**) Optical images of paperclip helical coil sensors under stretching, bending and twisting.

The manufacturing process of the circular needle spiral coil is depicted in Figure 1a. Initially, a silicone rubber tube of suitable length is selected for the experiment and immersed in an alcohol solution to cleanse and eliminate any contaminants present both inside and on the surface. Following the cleaning procedure, the tube is dried in an oven for 10 min, with the oven temperature maintained at 25 °C, utilizing the air circulation feature to facilitate the removal of residual water droplets within the tubes. Subsequently, liquid metal is injected into the silicone tube, with copper wire serving as the electrode, and the port is sealed using epoxy resin adhesive. The encapsulated silicone tube is then bent into the shape of a paperclip using a 3D printing mold. The A and B components of PDMS adhesive are weighed in a 10:1 ratio, mixed thoroughly, and placed in a vacuum chamber to remove air bubbles. The prepared PDMS adhesive is added to the mold for sample encapsulation and then placed on a heating table at 80 °C for curing over a period of 1 h. Once the sample is fully cured, it is maintained in a clean state. After returning to room temperature, the sample is removed from the mold for further testing.

The samples produced exhibit exceptional mechanical flexibility, allowing them to be stretched, twisted, wound, bent, and knotted without sustaining damage. As illustrated in Figure 1b–d, these samples can endure various deformations, including stretching, bending, and twisting, and rapidly recover their original shape without fracturing, thereby demonstrating superior mechanical properties.

### 2.3. Performance Testing

**Figure 2:**
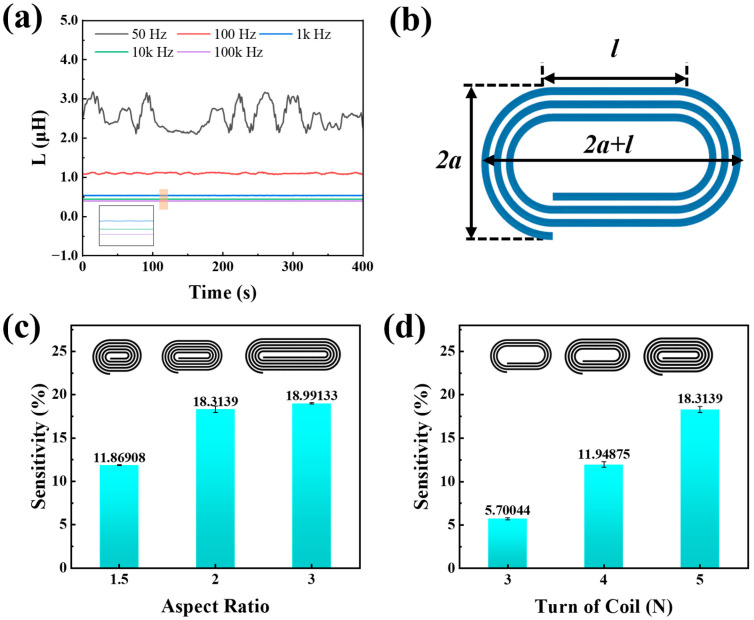
Definition and optimization of sensor parameters. (**a**) Inductance stability under different test frequencies. (**b**) Definition of structural parameters of paperclip helical coil sensor. (**c**) Strain sensitivity under different aspect ratios. (**d**) Strain sensitivity under different numbers of turns.

In this experiment, the TH2830 digital bridge from Changzhou Tonghui Electronics Co., Ltd., in Dongguan, Guangdong, China, is employed to measure the inductance of the coil. Given that actual inductors are non-ideal components characterized by internal winding resistance and core losses, among other factors, they necessitate representation through equivalent circuits. The digital bridge offers two methods for inductance measurement: the parallel equivalent model and the series equivalent model. Due to the coil’s characteristic of having a small inductance, the series measurement mode is selected. Using a coil with a transverse ratio of 2 and 4 turns as an example, the measurement frequency of the digital bridge is varied, and the stable inductance of the coil is measured over a period of 400 s, as depicted in Figure 2a. During the process of increasing the measurement frequency from 50 Hz to 100 kHz, significant fluctuations in inductance are observed, with the least stability occurring at 50 Hz. As the frequency increases, the inductance stability improves, reaching optimal levels at 10 kHz and 100 kHz, where changes are negligible. Consequently, 10 kHz was selected as the measurement frequency for this experiment. At this frequency, self-harmonic resonance is also precluded (Note S1, Supporting Information).

## 3. Results and Discussion

### 3.1. Theoretical Analysis

When current passes through a coil, a magnetic field is generated around it. The size of the inductance reflects how much magnetic field energy the coil can store. In this study, the length of the straight section of the sensor is denoted as l, while the curved section comprises two semi-circles with a radius of a (Figure 2b). Consequently, the total length of the coil is 2a+l, and its width is 2a. The overall inductance of the coil is the sum of the self-inductance and mutual inductance of each turn within the conductive channel. Utilizing a segmented magnetic coupling model, the coil is divided into a linear segment and a semi-circular segment. Employing principles from electromagnetic theory and engineering, the self-inductance and mutual inductance of each segment are approximately calculated [51]. According to Biot-Savart’s law [52] and Grover’s segmented inductance formula [53], the self-inductance of the linear segment is determined as

(1) $$L_{\mathrm{line}}=\frac{\mu_{0}l}{\pi }\left(\ln\frac{2l}{w}-1\right)$$

The semicircular section can be equated to a concentric toroidal coil [53,54]. By assuming radial homogeneity of the toroidal magnetic field, the classical formula for toroidal inductance is derived as

(2) $$L_{c}=\mu_{0}a\left(\ln\frac{8a}{w}-2\right)$$

So, the self-inductance of each coil turn is

(3) $$L_{i}=\frac{\mu_{0}l}{\pi }\left(\ln\frac{2l}{w}-1\right)+\mu_{0}a\left(\ln\frac{8a}{w}-2\right)$$

Similarly, the mutual inductance between adjacent turns is also divided into a straight-line segment and a semi-circular part. The mutual inductance between adjacent turns is calculated as

(4) $$M_{i,j}=\frac{\mu_{0}l}{\pi }\left(\ln\frac{2l}{d}-1\right)+\frac{\mu_{0}a}{2}g\left(\left|i-j\right|\right)$$

(5) $$L=\sum L_{i}+\sum M_{i,j}$$

In the aforementioned formula, w denotes the radius of the liquid metal pipe, d represents the spacing between each turn of the wire, l is the length of the straight segment of the wire, and a signifies the radius of the semi-circular segment. The exponential attenuation function $g\left(\left|i-j\right|\right)=e^{-\alpha (\left|i-j\right|-1)}\;(\alpha =0.5)$ is the mutual inductance attenuation function of concentric rings in the semi-circular segment [53]. For a coil characterized by an aspect ratio of 2 and comprising five turns, the theoretical inductance, as determined through Equations (1)–(5), is approximately 448.08 nH. However, experimental measurements indicate an initial inductance of 524.65 nH, resulting in a theoretical error of 14.59%. This discrepancy primarily arises because the theoretical calculations consider only the pure inductance, whereas the actual measurements, conducted using a digital bridge, capture the equivalent inductance. The equivalent inductance accounts for the minor magnetic coupling between the copper wire electrode and the coil ends, a factor not included in the theoretical model, thereby leading to a higher measured inductance value.

Based on prior studies, it has been established that the application of uniaxial tensile strain (ε) along the longitudinal direction (l) of a coil results in a more pronounced effect on the conductor’s length than on its width. During the deformation process, the variation in the straight segments of each coil turn is substantially greater than that in the curved sections, predominantly influencing the inductance changes in the coil. As the degree of tensile strain increases, the spacing between the straight wire segments decreases, leading to an increase in both mutual inductance and overall inductance [48]. Consequently, the inductance of the coil is observed to increase with higher strain levels. Furthermore, when a compressive test is performed on the sensor, as corroborated by relevant literature [41], there is an enhancement in mutual inductance and magnetic flux, which collectively contribute to an increase in the coil’s total inductance.

### 3.2. Parameter Optimization

We calculated the sensitivity δ of the coil to study the influence of the sensor’s structural parameters on the variation in its inductance. δ represents the change in electrical signal per unit deformation of the strain sensor:

(6) $$\delta =\frac{\bar{L}-\bar{L_{0}}}{\bar{L_{0}}\varepsilon }$$

*L*_0_ denotes the initial inductance, whereas *L* represents the sensor inductance measured after each test. The symbol ε indicates the degree of strain. In this study, we fabricated multiple sensors to examine the influence of coil structural parameters on their sensitivity. According to the existing literature, pipes with smaller diameters exhibit superior flexibility when constructed from the same material. Consequently, we selected a silicone tube with dimensions of 0.3 mm × 0.8 mm, the smallest diameter available from Shanghai Xinrong Rubber and Plastic Technology Products Co., Ltd., in Shanghai, China, to serve as the conductive path for the sensor. The sensor’s width is specified as 10 mm to ensure it is sufficiently compact for application on the relatively narrow regions of the finger. Within this 10 mm range, a coil with a maximum of five turns can be accommodated. To achieve a sensor with the optimal strain coefficient, we produced two sets of samples by changing the aspect ratio and the number of turns. The aspect ratio is defined as (2a+l)/2a. The first set consists of planar coils with a uniform number of turns (five turns) but varying transverse and aspect ratios (3:2, 2:1, 3:1). The second set maintains a constant aspect ratio (2:1) while varying the number of turns (3, 4, 5).

The sensors are subjected to strains ranging from 0% to 80%, and their resulting sensitivities are compared. According to the influence of the aspect ratio on sensitivity, as depicted in Figure 2c, it is observed that an increase in the aspect ratio from 1.5 to 3 results in a monotonically increasing trend in sensitivity. However, the increase in sensitivity is significantly more pronounced when the aspect ratio increases from 1.5 to 2 compared to the increase from 2 to 3. As elucidated in the preceding theoretical analysis, variations in inductance are predominantly ascribed to the presence of parallel straight conductors. The sensitivity of the sensor is augmented by the length of its parallel straight conductor. Owing to their structural configuration, an increased aspect ratio leads to a greater number of parallel straight conductors within the coil, thereby enhancing the sensitivity. However, the elongation of the coil shape also exacerbates stress concentration during mechanical deformation tests, potentially compromising structural reliability. The influence of the number of coil’s turns on sensor sensitivity is illustrated in Figure 2d. The findings demonstrate that as the number of turns increases, sensitivity consistently rises without evident coupling saturation. Notably, when the number of turns, N, reaches 5, the sensitivity attains the peak within the test range. This has resulted in notable alterations in the mutual inductance among various components of the sensor. In summary, the experimental findings indicate that the sensor achieves optimal sensitivity when the number of turns is set to five and the aspect ratio is maintained at two.

### 3.3. Performance of Strain Sensing

**Figure 3:**
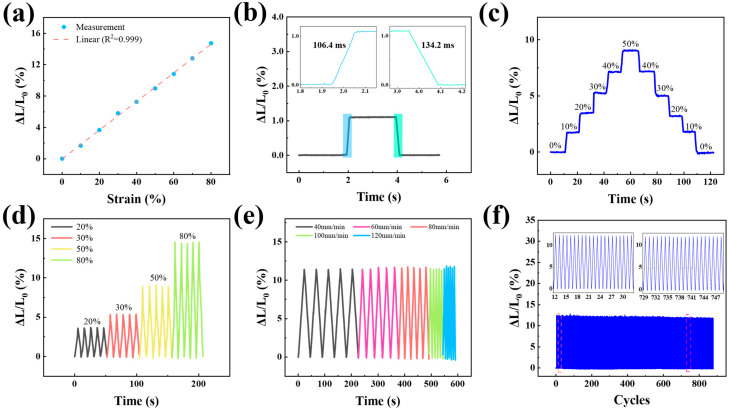
A variety of tensile tests. (**a**) Relative inductance changes were measured under tensile strain. (**b**) The sensor’s response and recovery times were evaluated at 1.1% tensile strain. (**c**) Inductance stabilization was demonstrated by maintaining the strain for 10 s under different conditions. (**d**) Changes in relative inductance were assessed at various cyclic strains. (**e**) Relative inductance variations were recorded during cyclic stretch-release tests at 60% strain at speeds of 40, 60, 80, 100, and 120 mm/min. (**f**) The sensor’s stability was tested over 800 stretch-release cycles at 60% strain.

To assess the sensor’s performance, a series of tensile and release tests were performed, beginning with a tensile test depicted in Figure 3a, where the sensor was gradually stretched to 80% strain. The resulting measurement curve demonstrates an approximately linear trend ($R^{2}=0.999$), with the inductance increasing by 14.64% at 80% strain compared to the initial value. Figure 3b shows that when a 1.1% strain is applied to the sensor and maintained for a brief period (2 s) before release, the sensor exhibits rapid response and recovery times of 106.4 ms and 134.2 ms, respectively. Furthermore, the sensor exhibited a stable inductance value with minimal fluctuations across various strain stages, ranging from 0% to 50% and then returning to 0%, with increments of 10% per strain stage, thereby demonstrating excellent numerical consistency. Figure 3d illustrates the relative changes in inductance of the sensor during tensile and contraction cycles at different strain levels, highlighting the variations in inductance and the sensor’s stable response under varying tensile strain conditions. Figure 3e presents the relative inductance changes when the sensor is cyclically stretched by 60% at speeds of 40, 60, 80, 100, and 120 mm/min. The similarity in relative inductance changes across different stretching frequencies under the same strain level suggests a negligible dependence on stretching frequency. As depicted in Figure 3f, the sensor can endure over 800 cycles of stretching and releasing at a speed of 80 mm/min and a strain of 60%. These results indicate an ideal long-term service life and collectively confirm the exceptional performance of our sensors under both high- and low-strain conditions.

**Figure 4:**
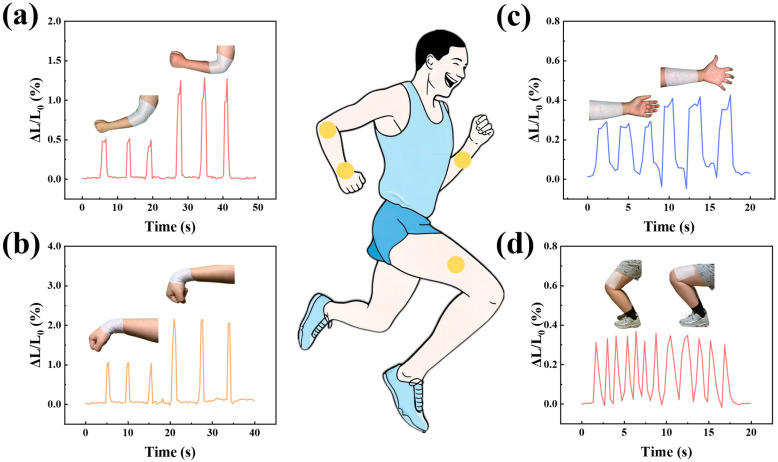
The application of sensors to monitor human physiological activities at various anatomical sites includes (**a**) detecting variations in inductance levels during elbow flexion; (**b**) tracking changes in relative inductance associated with wrist movements; (**c**) monitoring inductance signal fluctuations resulting from arm muscle contractions and relaxations during palm opening and closing; and (**d**) observing changes in inductance signals corresponding to leg muscle activity during squatting.

Leveraging the exceptional strain sensing capabilities of the sensor, we employed it to detect the bending angles of various joints in the human body, as well as the tension and relaxation states of muscles. Sensors were meticulously positioned on the wrists, arms, elbows, and leg muscles of the participants, enabling continuous tracking of movement patterns corresponding to their activities. As illustrated in Figure 4a, the sensor was affixed to the elbow joint of the participants, allowing for precise monitoring of arm flexion degrees. Notably, when the arm is flexed at a 90° angle, the inductive output reaches its optimal level. Conversely, when the arm is bent to a 45° angle, the response diminishes. Each state was repeated three times, demonstrating the inductive signal’s robust stability. Additionally, affixing the sensor to the wrist (Figure 4b) effectively detects the degree of wrist curvature. The brachioradialis muscle, located on the lateral aspect of the forearm, plays a crucial role in human motor function, joint stability, and daily activities. Alterations in the state of the brachioradialis muscle occur when the palm is open and relaxed. As depicted in Figure 4c, a sensor was positioned on the forearm of a volunteer, and variations in palm opening resulted in signals of markedly different intensities. Additionally, it was observed that the half-squat and full-squat positions induce changes in the state of the leg muscles (Figure 4d). By placing a sensor on the thigh and monitoring muscle signal variations across different squat positions, it was determined that the signal frequency was relatively low in the half-squat position and relatively high in the full-squat position, although the signal intensity remained comparable. In summary, our application of sensors to the human body, which translates subtle physiological changes into detectable signals, underscores the significant potential of sensors for applications in human body analysis.

### 3.4. Pressure and Position Sensor

**Figure 5:**
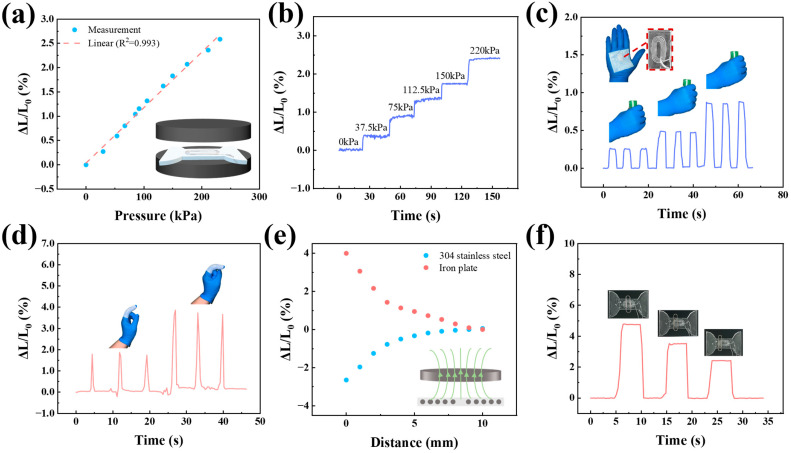
Evaluation and implementation of pressure and non-contact distance sensing properties of sensors. (**a**) Analysis of inductance signals for the sensor across an extensive pressure range (0 to 230 kPa). (**b**) Examination of the sensor’s response to incremental pressure variations between 0 and 220 kPa. (**c**) Assessment of grip strength using sensors embedded in the palm. (**d**) Observation of the index finger’s curvature. (**e**) Measurement of the distance between various metal plates utilizing sensors. (**f**) Determination of the paperclip’s position.

In addition to its application as a strain sensor, the device is also capable of functioning as a pressure sensor. As illustrated in Figure 5a, the sensor exhibits a commendable linear response across a broad pressure range of up to 230 kPa, achieving an $R^{2}$ value of 0.992, indicative of its exceptional linear pressure detection capabilities. Figure 5b depicts the variations in the inductive signal as incremental pressure is applied, revealing the sensor’s robust stability. The pressure-sensing performance of the aforementioned sensors underscores their extensive operational range without compromising performance, as well as their high repeatability.

We have integrated the sensor into a glove, enabling the detection of gesture information and palm grip strength. The signal strength is influenced by the degree of finger bending. Variations in the force exerted by the palm when grasping an object result in corresponding changes in pressure on the sensor, thereby altering the signal strength to varying degrees.

The sensor demonstrates exceptional capabilities in non-contact sensing of metal distances. When a non-ferromagnetic 304 stainless steel plate and a ferromagnetic iron plate were positioned parallel to the sensor and incrementally moved away, as illustrated in Figure 5e, it was observed that within a 10 mm range, the 304 stainless steel plate led to a 3% reduction in sensor inductance, whereas the iron plate resulted in a 4% increase in sensor inductance. These opposite signal changes are attributed to the substantial disparity in their magnetic permeability. Specifically, iron plates, characterized by high magnetic permeability $\left(\mu \;>\;\mu_{0}\right)$, act as magnetic media that enhance the coil’s magnetic field, leading to an increase in the sensor’s inductance value. Conversely, 304 stainless steel is a non-magnetic conductor with a magnetic permeability approximately equal to that of a vacuum $\left(\mu \;\approx \;\mu_{0}\right)$ and possesses high electrical conductivity. This results in the induction of eddy currents that generate magnetic fields opposing the original field, thereby decreasing the sensor’s inductance value.

As illustrated in Figure 5f, the sensor exhibits excellent pressure and distance sensing characteristics. When a paperclip is positioned at different locations on the sensor, the signal intensity varies accordingly. The signal is strongest when the paperclip is closest to the center of the sensor highlighting its significant potential for distance sensing applications.

### 3.5. Process Comparison

Table 1 summarizes recent advancements in the development of inductive sensors for liquid metal materials. Traditionally, the fabrication of microchannel molds necessitates the use of high-precision instruments, a process characterized by its complexity, difficulty, and potential for material wastage. In contrast, our sensor manufacturing process eliminates the need for precision molds, thereby minimizing material waste and significantly reducing both equipment investment and material loss. Furthermore, during the material filling phase, the injection of liquid metal into smooth, fine silicone tubes enhances the stability of sample quality and facilitates rapid preparation across various scenarios. This approach offers greater versatility and scalability in the customized production of flexible electronic devices, wearable sensors, and related applications. The origin of the flexible substrates mentioned in the references is reflected in Supporting Information Note S3.

## 4. Conclusions

This study introduces a novel multifunctional flexible inductive sensor utilizing liquid metal. Notably, the sensor features a distinctive paperclip-type thin-layer helical structure and employs a simplified manufacturing process, offering an innovative approach to flexible inductive sensing. The sensor is constructed by encapsulating a fine silicone tube filled with liquid metal within a PDMS adhesive. By leveraging the similar Young’s modulus of the silicone tube and PDMS adhesive, the design effectively minimizes the risk of leakage, such as channel collapse or insulation layer rupture due to stress concentration. Furthermore, this approach eliminates the need for high-precision sacrificial molds or costly equipment, thereby significantly reducing manufacturing complexity and cost. This facilitates rapid production and customization for various applications. The experimental results indicate that the sensor exhibits exceptional linearity in tensile sensing ($R^{2}=0.999)$, and maintains consistent performance even after more than 800 tensile cycles, thereby demonstrating high reliability and durability. In pressure sensing, the sensor delivers a stable response over a broad range of 0 to 230 kPa, with great linearity ($R^{2}=0.992$). For non-contact distance sensing, the sensor effectively differentiates between ferromagnetic and non-ferromagnetic metal materials within a 10 mm range. Furthermore, the sensor has been successfully employed in monitoring various human physiological activities, such as muscle contraction and joint bending angle detection, as well as in gesture and grip strength recognition and non-contact metal distance perception. These capabilities underscore its practical applicability in domains such as health monitoring, human–computer interaction, and flexible robotics. In conclusion, the inductive sensing sensor introduced in this study, characterized by its straightforward and cost-effective manufacturing process, multifunctional integration capabilities, and exceptional sensing performance, offers a viable pathway for the large-scale deployment of flexible electronic devices. This underscores its significant potential within the domain of flexible wearable technology.

## Figures and Tables

**Table 1:** A review of the performance specifications of several types of inductive sensors for liquid metal materials.

Layers	Turns	Function	Workmanship	Materials	Sensitivity	Reference
1	11	Strain	Print coil patterns on flexible substrates by scraping	Ecoflex 0030	0.371	[40]
3	5	Pressure	Sacrifice low-melting-point alloys to form microchannels	Ecoflex 0030 PDMS	0.369 kPa^−1^ 0.0657 kPa^−1^	[42]
1	15	Strain	3D printed microchannels	Ecoflex 0030	0.64	[48]
2	5	Pressure	Sacrifice low-melting-point alloys to form microchannels	PDMS@Fe (Fe:PDMS = 1:1)	0.0545 kPa^−1^	[55]
1	5	Pressure and strain	Commercial silicone tubes are used as microchannels	PDMS and Silicone tube	0.0113 kPa^−1^ 0.183	This work

## Data Availability

The original contributions presented in this study are included in the article/supplementary material. Further inquiries can be directed to the corresponding author.

## Supplementary Materials

The following supporting information can be downloaded at https://www.mdpi.com/article/10.3390/mi16080965/s1, Note S1: Measurement frequency selection; Note S2: Pressure test Instructions; Note S3: Origin of the Materials in Table 1; Figure S1: Physical pictures of pressure tests. Reference [56] is cited in Supplementary Materials.
