# Supplementary material for: Paperclip-Type Flexible Inductive Sensor Based on Liquid Metal Coils for Simple Fabrication and Multifunctional Applications
Source: Micromachines (Basel). 2025 Aug 21;16(8):965. doi: 10.3390/mi16080965 (PMC12388588; doi:10.3390/mi16080965)
Supplement: Supplementary file 1 [file micromachines-16-00965-s001.zip › micromachines-3825715-supplementary.pdf]

Supporting Information

Paperclip-Type Flexible Inductive Sensor Based on Liquid Metal Coils for Simple Fabrication and Multifunctional Applications

Xun Sun [1,2], Kaixin Li [1,2], Zifeng Zhang [1,2], Linling Xiang [1,2], Yihao Zhou [1,2], and Bin Sheng [1,2,*]

1 School of Optical Electrical and Computer Engineering, University of Shanghai for Science and Technology, Shanghai 200093, China; 2235052705@st.usst.edu.cn (X.S.); 233350670@st.usst.edu.cn (K.L.); 233350727@st.usst.edu.cn (Z.Z.); 233350619@st.usst.edu.cn (L.X.); 242200346@st.usst.edu.cn (Y.Z.);

2 Shanghai Key Laboratory of Modern Optical Systems, Engineering Research Center of Optical Instruments and Systems, Shanghai 200093, China

* Correspondence: bsheng@usst.edu.cn

Note S1: Measurement frequency selection

Our coil, with an inductance of approximately 1 μH, measures 20 mm in length and 10 mm in width, and consists of 5 turns. These dimensions confer characteristics of a thin layer, few turns, and compact size. Furthermore, the use of PDMS and silicone, both materials with low dielectric constants, contributes to the reduction of parasitic capacitance. According to established electromagnetic theories [1], the parasitic capacitance for coils of this specification typically ranges from 1 pF to 20 pF. For precautionary purposes, we assume the upper limit value of C = 20 pF. Therefore, the self-resonant frequency is obtained.

$$f = \frac{1}{2\pi\sqrt{LC}} = 35.6MHz$$

The selected measurement frequency of 10 kHz is significantly lower than the resonant frequency, thereby eliminating the possibility of resonant interference due to parasitic capacitance.

Note S2: Pressure test Instructions

The positive pressure test was employed, wherein the area of the applied pressure head significantly exceeds that of the liquid metal coil within the sensor, thereby ensuring uniform pressure distribution. Upon application of a pressure of 230 MPa, the sensor's thickness decreases from its initial 2 mm to 1.77 mm. Given that PDMS adhesive is an incompressible elastic material (with a Poisson's ratio of v=0.5), the sensor's volume remains constant during deformation. Consequently, calculations indicate that the sensor's width increases to 10.575 mm and its length to 21.15 mm. At this juncture, the lateral strain reaches 5%, with a corresponding strain sensitivity of 0.9%, which is considerably lower than the inductance change induced by 230 MPa. Thus, it can be concluded that under conditions of small deformations, pressure exerts a predominant influence.

Note S3: Origin of the Materials in Table 1

In reference [40], Ecoflex 0030 is from Beijing Tiantong Huayi Co. Ltd., in Beijing, China. In reference [42], Ecoflex 0030 is from Smooth-On, USA. In reference [48], PDMS is Dow Corning Sylgard 184 Silicone Elastomer, and there are no reports about Ecoflex. In reference [55], PDMS is Dow Corning Sylgard 184 Silicone Elastomer.

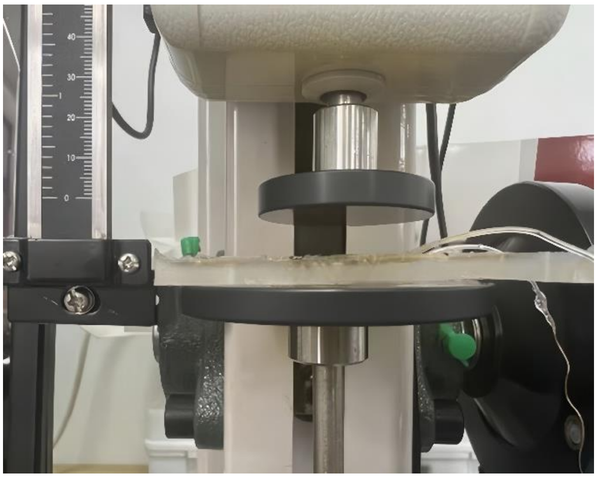

Figure S1. Physical pictures of pressure tests

1. Wheeler, H.A. Formulas for the Skin Effect. *Proceedings of the IRE* **1942**, *30*, 412–424, doi:10.1109/JRPROC.1942.232015.
